# Preparative Separation of Alkaloids from *Picrasma quassioides* (D. Don) Benn. by Conventional and pH-Zone-Refining Countercurrent Chromatography

**DOI:** 10.3390/molecules19078752

**Published:** 2014-06-25

**Authors:** Qinghai Zhang, Xikai Shu, Feng Jing, Xiao Wang, Changhu Lin, Aiqin Luo

**Affiliations:** 1 Department of Biochemical Engineering, School of Life Science, Beijing Institute of Technology, Beijing 100081, China; E-Mail: zhqh8@163.com; 2 Guizhou Academy of Testing and Analysis, Guiyang 550002, China; 3 TCM Process Control Research Center, Shandong Analysis and Test Center, Shandong Academy of Sciences, 19 Keyuan Street, Jinan 250014, China; E-Mails: shuxikai5@163.com (X.S.); gaoaixiangjf@163.com (F.J.); 4 The Key Laboratory of Chemistry for Nature Products, Guizhou Province and Chinese Academy of Sciences, Guiyang 550002, China; E-Mail: linchanghu79@sina.com

**Keywords:** alkaloids, *Picrasma quassioides*, pH-zone-refining countercurrent chromatography, separation

## Abstract

Two high-speed countercurrent chromatography (HSCCC) modes were compared by separation of major alkaloids from crude extract of *Picrasma quassioides*. The conventional HSCCC separation was performed with a two-phase solvent system composed of petroleum ether–ethyl acetate–methanol–water (5:5:4.5:5.5, v/v/v/v) with 200 mg loading. pH-Zone-refining CCC was performed with two-phase solvent system composed of petroleum ether–ethyl acetate–*n*-butanol–water (3:2:7:9, v/v/v/v) where triethylamine (10 mM) was added to the upper organic stationary phase and hydrochloric acid (5 mM) was added to the lower aqueous phase with 2 g loading. From 2 g of crude extract, 87 mg of 5-methoxycanthin-6-one (a), 38 mg of 1-methoxy-β-carboline (b), 134 mg of 1-ethyl-4,8-dimethoxy-β-carboline (c), 74 mg of 1-ethoxycarbonyl-β-carboline (d), 56 mg of 1-vinyl-4,8-dimethoxy-β-carboline (e) and 26 mg of 1-vinyl-4-dimethoxy-β-carboline (f) were obtained with purities of over 97.0%. The results indicated that pH-zone-refining CCC is an excellent separations tool at the multigram level.

## 1. Introduction

*Picrasma quassioides* (D. Don) Benn., referred to as “Kumu” in China, is widely distributed in most areas of mainland China. The branches of this plant are used as a traditional folk medicine for the treatment of a variety of diseases such as gastroenteritis, hypertension, cancer and snakebites [1,2]. The major active compounds in *Picrasma quassioides* are β-carbolines and canthin-6-one alkaloids which were reported to inhibit cAMP phosphodiesterase [3], and have anti-inflammatory, anti-hypertension and antiviral activities [2,4].

Because of these important activities and wide usage, large quantities of pure compounds are needed for further modern pharmacological and metabolic studies, as well as in the quality control of this traditional Chinese medicine and its products. Unfortunately, most of the alkaloids of *Picrasma quassioides* are not commercially available as preparative separation and purification of these bioactive compounds by classical methods are very difficult, tedious and usually require multiple chromatography steps. Thus, it is necessary to develop a rapid and efficient method to separate these alkaloids in larger quantites.

High-speed countercurrent chromatography (HSCCC) and pH-zone-refining CCC, introduced by Ito [5,6], have been widely used in the isolation of natural products [7,8,9,10,11]. HSCCC is a support-free liquid-liquid chromatographic technique which allows complete recovery of samples and the separation relies on partition of a sample between two immiscible liquid phases [5]. pH-Zone-refining CCC is a special large-scale preparative technique for separating ionizable compounds according to their pKa values and hydrophobicities [6]. In addition, the method provides many special features by yielding highly concentrated fractions, concentrating minor impurities for detection, and allowing the separation to be monitored by the pH of the effluent when no chromophores are present. Alkaloids are thus good candidates for pH-zone-refining CCC purification. A paper about a convenient HSCCC method to isolate alkaloids from *Picrasma quassioides* only obtained three compounds [12]. No report has been found on the use of pH-zone-refining CCC for the isolation and purification of alkaloids from *Picrasma quassioides*. In the present paper, HSCCC in its two forms, conventional and pH-zone-refining CCC, was successfully applied to the separation of alkaloids from crude extract of *Picrasma quassioides*.

## 2. Results Discussion

### 2.1. Optimization of HPLC Conditions

A HPLC method was optimized to ensure baseline separation of target compounds and impurities. Different mobile phase compositions (methanol–water, acetonitrile–water) were screened. To enhance the separation resolution, acids, including acetic acid, formic acid acid or phosphoric were added to the mobile phase. After experiments, acetonitrile and water containing 0.2% phosphoric acid were chosen as the eluting solvent system.

### 2.2. Separation of the Crude Extract of Picrasma quassioides by Conventional HSCCC

The solvent systems were selected according to the partition coefficient (*K*_D_) of target compounds. A series of solvent systems (Pet–EtOAc–MeOH–H_2_O) were tested, as shown in Table 1. The *K*_D_ of Pet–EtOAc–MeOH–H_2_O (5:5:4.5:5.5, v/v/v/v) was suitable, so the conventional HSCCC studies were carried out with two-phase solvent system composed of Pet–EtOAc–MeOH–H_2_O (5:5:4.5:5.5, v/v/v/v). The experiments showed that a good separation was achieved when Pet–EtOAc–MeOH–H_2_O (5:5:4.5:5.5, v/v/v/v) was used. 

**Table 1:** The partition coefficient (*K*) values in both acidic and alkaline of different systems.

Solvent System (Pet–EtOAc–MeOH–H_2_O) (v/v)	*K*_D__a_	*K*_D__b_	*K*_D__c_	*K*_D__e_	*K*_D__f_	*K*_D__g_
5:5:5:5	0.18	0.26	1.34	0.96	0.64	1.72
5:5:4.5:5.5	0.24	0.48	2.24	1.82	1.45	2.68
5:5:4:6	0.47	0.96	3.12	2.41	1.96	3.44

**Figure 1:**
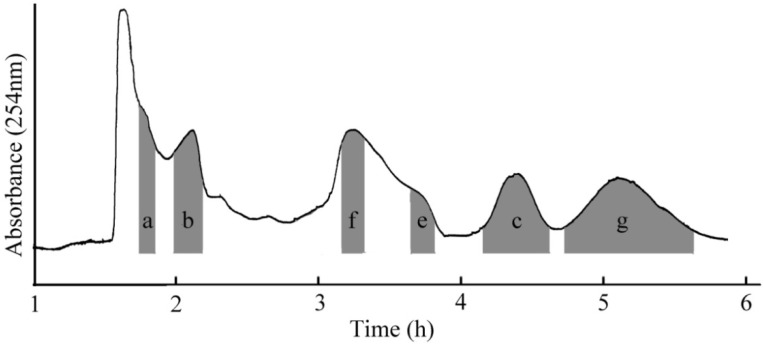
Conventional HSCCC chromatogram of separation crude extracts from *Picrasma quassioides*; Conditions: Two-phase solvent system: Pet–EtOAc–MeOH–H_2_O (5:5:4.5:5.5, v/v/v/v); the retention of the stationary phase: 46%; flow rate: 2 mL/min; detection wavelength: 254 nm; revolution speed: 850 rpm; sample size: 200 mg.

The chromatogram (Figure 1) showed that six alkaloid compounds were successfully separated using the optimized solvent system. The retention of the stationary phase was 64%, and the separation time was about 6 h in each separation run. 

**Figure 2:**
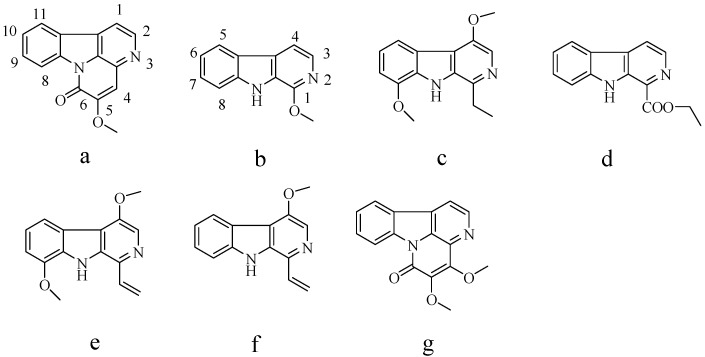
Chemical structures of alkaloids from *Picrasma quassioides*.

Six compounds were obtained (Figure 2): 5-methoxycanthin-6-one (a, 4 mg), 1-methoxy-β-carboline (b, 8 mg), 1-ethyl-4,8-dimethoxy-β-carboline (c, 13 mg), 1-vinyl-4,8-dimethoxy-β-carboline (e, 8 mg), 1-vinyl-4-dimethoxy-β-carboline (f, 11 mg), and 4,5-dimethoxycanthin-6-one (g, 14 mg) with purities of 93.4%, 95.7%, 96.1%, 95.4%, 97.7%, and 97.1%, respectively, which were determined by using a normalization method (the corresponding HPLC chromatogram is not shown). Attempts to separate larger quantities of this mixture by conventional HSCCC failed mainly due to the poor retention of the stationary phase and resulted in a poor resolution.

### 2.3. Separation of the Crude Extract of Picrasma quassioides by pH-Zone-Refining CCC

In order to separate a large amount of target compounds, pH-zone-refining CCC was used to separate alkaloids from *Picrasma quassioides* because of its large capacity which can be 2–10 g. According to the rules for selection of solvent systems described in the references [8,9], four solvent systems composed of Pet–EtOAc–MeOH–H_2_O at volume ratios of 5:5:1:9 and 5:5:0:10, Pet–EtOAc–*n*-butanol–H_2_O at volume ratios of 5:5:1:9 and 3:2:7:9 were tested. The elution ability of this four solvent system was weak. After trying the above solvent systems, the first three solvent systems eluted too fast and the solvent system Pet–EtOAc–*n*-butanol–H_2_O (3:2:7:9, v/v/v/v) was found to be suitable for the preparative separation.

**Figure 3:**
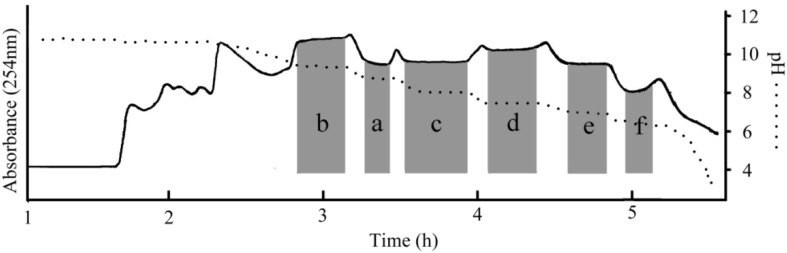
pH-zone-refining CCC chromatogram of preparative separation of alkaloids compounds from *Picrasma quassioides*; Conditions: Two-phase solvent system: petroleum ether–ethyl acetate–*n*-butanol–water at volume ratios (3:2:7:9, v/v/v/v); the retention of the stationary phase: 34%; 10 mM TEA in stationary phase and 5 mM HCl in lower phase; flow rate: 2 mL/min; detection wavelength: 254 nm; revolution speed: 850 rpm; sample size: 2.0 g.

**Figure 4:**
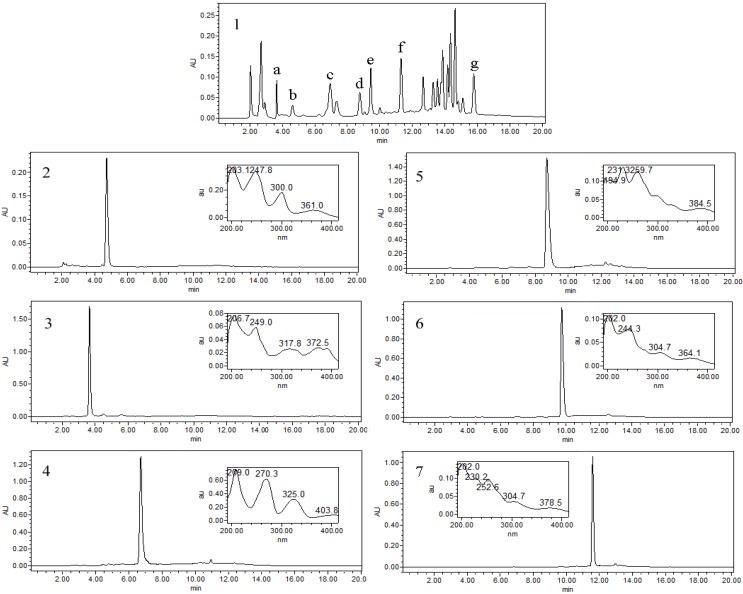
HPLC chromatograms: (**1**) Crude extracts from *Picrasma quassioides*; (**2**) compound **a**: 5-methoxycanthin-6-one; (**3**) compound **b**: 1-methoxy-β-carboline, 1-ethyl-4; (**4**) compound **c**: 8-dimethoxy-β-carboline; (**5**) compound **d**: 1-ethoxycarbonyl-β-carboline; (**6**) compound **e**: 1-vinyl-4,8-dimethoxy-β-carboline; (**7**) compound **f**: 1-vinyl-4-dimethoxy-β-carboline.

Figure 3 shows a typical chromatogram for separation of 2.0 g of crude extract by pH-zone-refining CCC. The retention of the stationary phase was 34%, and the total separation time was about 5.5 h. Alkaloids were eluted as an irregular rectangular peak where six absorbance plateaus were observed. The pH measurement of collected fractions also revealed six flat pH zones which respectively correspond to the above absorbance plateaus, which indicted that the six alkaloids were successfully separated. The impurities formed multiple peaks in the front and the back of the main peak. From 2.0 g of the crude extract in one run, 87 mg of 5-methoxycanthin-6-one (a), 38 mg of 1-methoxy-β-carboline (b), 76 mg of 1-ethyl-4,8-dimethoxy-β-carboline (c), 74 mg of 1-ethoxy- carbonyl-β-carboline (d), 56 mg of 1-vinyl-4,8-dimethoxy-β-carboline (e) and 26 mg of 1-vinyl-4-dimethoxy-β-carboline (f) were obtained in one step separation with purities of 98.1%, 98.2%, 98.4%, 98.7%, 99.1%, and 98.1%, respectively, as determined by HPLC (Figure 4).

Both the conventional and pH-zone-refining modes of CCC successfully performed the preparative separation of alkaloids from crude extract of *Picrasma quassioides*. Comparing these two modes of CCC, the elution order of target compounds was different, indicating that the elution principle was different between pH-zone-refining CCC according to their pKa values and hydrophobicities and HSCCC according to *K*_D_. As shown in Table 2. The results also clearly demonstrated that pH-zone-refining CCC had many advantages over conventional CCC. It gave an over 10-fold increase in sample-loading capacity, high purity, high yield, high recovery and high concentration of the collected fractions.

**Table 2:** Comparison between the two separation methods.

	HSCCC						pH-Zone-Refining CCC					
	a	b	c	e	f	g	a	b	c	d	e	f
Purity (%)	93.4	95.7	96.1	95.4	97.7	97.1	98.1	98.2	98.4	98.7	99.1	98.1
Yield (mg)	4	8	13	8	11	14	87	38	76	74	56	26
Recovery (%)	76	82	92	78	72	94	94	92	94	96	95	91
sample size	200 mg						2 g					

## 3. Experimental

### 3.1. Reagents and Materials

Chromatographic grade methanol (Tedia Company Inc., Fairfield, OH, USA) was used for HPLC analysis. Deionized water used in solutions and dilutions was treated with a Milli-Q water purification system (Millipore, Boston, MA, USA). HSCCC and extraction reagents were of analytic grades (Damao Chemical Factory, Tianjin, China). The triethylamine (TEA) and hydrochloric acid (HCl) (Laiyang Huagong, Laiyang, China) used for pH-zone-refining CCC were analytic grade. The *Picrasma quassioides* was purchased from a local drug store and identified by Dr. J. Li (College of Pharmacy, Shandong University of Traditional Chinese Medicine, Shandong, China).

### 3.2. Apparatus

The pH-zone-refining CCC in the present study is performed with a Model TBE-300A high-speed count-current chromatograph (Shanghai Tauto Biotech Co., Ltd., Shanghai, China) with three PTFE preparative coils connected in series (internal diameter of tube, 1.6 mm; total volume, 300 mL) and a 20 mL sample loop. The β-values of this preparative column ranged from 0.47 at the internal to 0.73 at the external (β = r/R, where r is the rotation radius or the distance from the coil to the holder shaft, and R (R = 7.5 cm) is the revolution radius or the distance between the holder axis and the central axis of the centrifuge). The system was also equipped with a Model NS-1007 constant-flow pump (Beijing Emilion Science & Technology, Beijing, China), a Model 8823A-UV detector (Beijing Emilion Science & Technology) at 254 nm and a Model 3057 portable recorder (Yokogawa, Sichuan Instrument Factory, Chongqing, China), a model UB-7 ph meter (Denver Instruments, Beijing, China).

The HPLC equipment used was including Waters 600 pump, Waters 600 controller and Waters 996 photodiode array detector (Waters, Milford, MA, USA). Evaluation and quantification were made on an Empower pro data handling system (Waters).

The identification of CCC peak fractions were carried out by electrospray ionization mass spectrometry (ESI-MS) on an Agilent 1100/MS-G1946 (Agilent, Santa Clara, CA, USA) and ^1^H-NMR spectra on a Varian-600 NMR spectrometer (Varian, Palo Alto, CA, USA).

### 3.3. Preparation of Crude Extract

Five Kg of dried *Picrasma quassioides* was ground into powder and extracted three times by the reflux method with 95% ethanol solution to which 10 mL HCl was added. After filtration, the extracts were combined and evaporated to dryness by rotary vaporization under reduced pressure. Then the residue was dissolved with 2 L of 1% HCl. The acidic extracts were made alkaline to pH 9.5 with NH_3_-water after exacted by petroleum ether. Then it was exacted by chloroform and evaporated to dryness. 54 g of crude alkaloids were obtained and the yield of crude alkaloids is 0.93%.

### 3.4. Separation Procedure

In the conventional HSCCC separation, the selected solvent systems of petroleum ether–ethyl acetate–methanol–water was thoroughly equilibrated in a separation funnel by repeated vigorous shaking at room temperature. The upper phase was used as the stationary phase, while the lower phase was used as the mobile phase. The sample solution was prepared by dissolving the crude extract in a 10 mL solution composed of the upper and lower phases (1:1, v/v) of the solvent system used HSCCC separation. The separation was initiated by filling the entire column with the stationary phase and then mobile phase was then pumped into the column at 2 mL/min while the column was rotated at 850 rpm in the combined head to tail elution mode. After the hydrodynamic equilibrium was reached, the sample solution was injected through the sample port. The effluent was monitored with the UV detector at 254 nm and the chromatogram was recorded. The whole process of separation occurred at room temperature. The fractions were collected according to the chromatogram and were analyzed by HPLC.

pH-Zone-refining CCC experiment was performed with a series of two-phase solvent systems composed of petroleum ether–ethyl acetate–methanol–water and petroleum ether–ethyl acetate–*n*-butanol–water, 10 mM TEA in upper phase and 5 mM HCl in lower phase. The sample solution was prepared by dissolving crude sample in 30 mL mixture of upper phase and lower phase (1:1, v/v). CCC separation was performed as follows: the multiplayer coiled column was firstly entirely filled with the upper phase (stationary phase). Then the apparatus was rotated at 850 rpm and the sample solution was injected into the column through sample loop. After that the lower phase (mobile phase) was pumped from the head of the column into the end at a flow rate of 2.0 mL/min. The effluent from the tail end of the column was continuously monitored with a UV detector at 254 nm and the chromatogram was recorded. Each peak fraction was manually collected according to the UV absorbance profile. The pH of each eluted fraction was measured with a pH meter. The fractions collected were 

After the separation was completed, retention of the stationary phase was measured by collecting the column contents into a graduated cylinder by forcing them out of the column with pressurized air The fractions collected were brought to dryness using a rotary evaporator under reduced pressure. The compounds separated by pH-zone-refining CCC were dissolved by water and extracted by chloroform to get rid of the salts.

### 3.5. HPLC Analysis and Identification of CCC Fractions

The crude sample and each peak fraction from CCC were analyzed by HPLC. The analysis was accomplished by a Waters SymmetryShield™ RP18 column (250 mm × 4.6 mm I.D., 5 μm) at room temperature. Acetonitrile and water (including 0.2% phosphoric acid) was used as the mobile phase in gradient elution mode as follows: acetonitrile: 0–20 min, 20% to 80%. The flow-rate of the mobile phase was 1.0 mL/min. The effluent was monitored at 254 nm by PAD.

### 3.6. Identification of Isolated Compounds

*5-Methoxycanthin-6-one* (**a**): Positive ESI–MS, *m*/*z* 250.8 [M+H]^+^. ^1^H-NMR (600 MHz, DMSO-*d*_6_) δ: 4.07 (3H, s, C_5_-OCH_3_), 7.51 (1H, d, *J* = 6.6, H-10), 7.54 (1H, t, *J* = 7.2 Hz, H-9), 7.87 (1H, t, *J* = 7.8 Hz, H-1), 8.08 (1H, d, *J* = 6.6 Hz, H-11), 8.24 (1H, d, *J* = 7.8 Hz, H-8), 8.78 (1H, d, *J* = 8.4 Hz, H-2). Compared with the data given in [13], compound (**a**) corresponded to 5-methoxycanthin-6-one.

*1-Methoxy-β-carboline* (**b**): Positive ESI–MS, *m*/*z* 198.9 [M+H]^+^. ^1^H-NMR (600 MHz, DMSO-*d*_6_) δ: 4.11 (3H, s, C_1_-OCH_3_), 7.41 (1H, t, *J* = 7.2 Hz, H-7), 7.75 (1H, d, *J* = 7.8 Hz, H-6), 7.80 (1H, d, *J* = 8.4 Hz, H-4), 8.43 (1H, d, *J* = 5.4 Hz, H-8), 8.48 (1H, d, *J* = 7.8 Hz, H-5), 8.61 (1H, d, *J* = 5.0 Hz, H-3), 10.04 (1H, s, -NH). Compared with the data given in [14], compound (**b**) corresponded to 1-methoxy-β-carboline.

*1-Ethyl-4,8-dimethoxy-β-carboline* (**c**): Positive ESI–MS, *m*/*z* 256.9 [M+H]^+^. ^1^H-NMR (600 MHz, DMSO-*d*_6_) δ: 1.54 (3H, t, C_1_-CH_3_), 3.17 (2H, q, C_1_-CH_2_), 4.09 (3H, s, C_8_-OCH_3_), 4.21 (3H, s, C_4_-OCH_3_), 6.98 (1H, d, *J* = 8.0 Hz, H-7), 7.21 (1H, t, *J* = 8.0 Hz, H-6), 7.90 (1H, d, *J* = 8.0 Hz, H-5), 7.96 (1H, s, H-3), 8.62 (1H, s, -NH). Compared with the data given in [15], compound (**c**) corresponded to 1-ethyl-4,8-dimethoxy-β-carboline.

*1-Ethoxy carbonyl-β-carboline* (**d**): Positive ESI–MS, *m*/*z* 240.9 [M+H]^+^. ^1^H-NMR (600 MHz, DMSO-*d*_6_) δ: 4.18 (3H, s, C_1_-COOCH_3_), 7.16 (1H, d, *J* = 7.8 Hz, H-8), 7.24 (1H, t, *J* = 7.8 Hz, H-7), 7.65 (1H, t, *J* = 7.8 Hz, H-6), 7.79 (1H, d, *J* = 7.2 Hz, H-5), 8.04 (1H, s, H-3), 10.65 (1H, s, C_4_-OH), 12.92 (1H, s, -NH). Compared with the data given in [16], compound (**d**) corresponded to 1-ethoxy carbonyl-β-carboline.

*1-Vinyl-4,8-dimethoxy-β-carboline* (**e**): Positive ESI–MS, *m*/*z* 254.8 [M+H]^+^. ^1^H NMR (600 MHz, DMSO-*d*_6_): δ: 4.24 (3H, s, C_8_-OCH_3_), 4.25 (3H, s, C_4_-OCH_3_), 5.64 (1H, q, H-9), 6.34 (1H, q, H-10), 6.96 (1H, d, *J* = 7.8 Hz, H-8), 7.31 (1H, q, H-10), 7.36 (1H, t, H-7), 7.70 (1H, t, H-6), 7.95 (1H, s, H-3), 8.65 (1H, s, -NH). Compared with the data given in [17], compound (**e**) corresponded to 1-vinyl-4,8-dimethoxy-β-carboline.

*1-Vinyl-4-dimethoxy-β-carboline* (**f**): ESI-MS: *m*/*z* 224.9 [M+H]^+^. ^1^H NMR (600 MHz, DMSO-*d*_6_): δ: 4.25 (3H, s, C_4_-OCH_3_), 5.64 (1H, q, H-9), 6.34 (1H, q, H-10), 6.96 (1H, d, *J* = 7.8 Hz, H-8), 7.31 (1H, q, H-10), 7.36 (1H, t, H-7), 7.70 (1H, t, H-6), 7.95 (1H, s, H-3), 8.65 (1H, s, -NH). Compared with the data given in [17], compound (**f**) corresponded to 1-vinyl-4-dimethoxy-β-carboline.

*4,5-Dimethoxycanthin-6-one* (**g**): ESI-MS: *m*/*z* 280.8 [M+H]^+^. ^1^H NMR (600 MHz, DMSO-*d*_6_): δ: 3.84 (3H, s, C_4_-OCH_3_), 4.25 (3H, s, C_4_-OCH_3_), 7.44 (1H, td, *J* = 11.8, 1.2 Hz, H-10), 7.64 (1H, td, *J* = 11.8, 1.2 Hz, H-9), 8.13 (1H, d, *J* = 7.8 Hz, H-1), 8.21 (1H, d, *J* = 11.3 Hz, H-11), 8.36 (1H, d, *J* = 11.3 Hz, H-8), 8.70 (1H, d, *J* = 7.2 Hz, H-2). Compared with the data given in [17], compound (**g**) corresponded to 4,5-dimethoxycanthin-6-one.

## 4. Conclusions

After comparing both conventional and pH-zone-refining CCC modes we report that pH-zone-refining CCC could separate multigram crude extracts while conventional HSCCC only can separate up to hundred milligram quantities of sample. The results indicate that pH-zone-refining CCC has many advantages over conventional CCC, such as an over 10-fold increase in sample-loading capacity, high purity, and high concentration of the collected fraction and it is an efficient technique to separate alkaloids from plants.
